# Unveiling the immune symphony: decoding colorectal cancer metastasis through immune interactions

**DOI:** 10.3389/fimmu.2024.1362709

**Published:** 2024-02-13

**Authors:** Ru He, Shangke Huang, Jiaan Lu, Lanqian Su, Xinrui Gao, Hao Chi

**Affiliations:** ^1^ Clinical Medical College, Southwest Medical University, Luzhou, China; ^2^ Department of Oncology, The Affiliated Hospital of Southwest Medical University, Luzhou, China; ^3^ Department of Oncology, Yongchuan Hospital of Traditional Chinese Medicine, Chongqing, China

**Keywords:** metastatic colorectal cancer, immunological characteristic, immune escape mechanisms, tumor microenvironment, TME, PD-L1, immunization checkpoints

## Abstract

Colorectal cancer (CRC), known for its high metastatic potential, remains a leading cause of cancer-related death. This review emphasizes the critical role of immune responses in CRC metastasis, focusing on the interaction between immune cells and tumor microenvironment. We explore how immune cells, through cytokines, chemokines, and growth factors, contribute to the CRC metastasis cascade, underlining the tumor microenvironment’s role in shaping immune responses. The review addresses CRC’s immune evasion tactics, especially the upregulation of checkpoint inhibitors like PD-1 and CTLA-4, highlighting their potential as therapeutic targets. We also examine advanced immunotherapies, including checkpoint inhibitors and immune cell transplantation, to modify immune responses and enhance treatment outcomes in CRC metastasis. Overall, our analysis offers insights into the interplay between immune molecules and the tumor environment, crucial for developing new treatments to control CRC metastasis and improve patient prognosis, with a specific focus on overcoming immune evasion, a key aspect of this special issue.

## 1 Background

Colorectal cancer (CRC), a major global health concern, recorded 1.93 million new cases and approximately 916,000 deaths in 2020, making it the second deadliest cancer worldwide (1, 2). A significant proportion of early-stage CRC patients (25%-50%) progress to metastasis, mainly affecting the liver, lungs, peritoneum, and distant lymph nodes, with a five-year survival rate for metastatic CRC at about 14% (3). The interaction between the immune system and tumor growth, involving phases of elimination, equilibrium, and escape, is crucial in CRC progression (4). Despite progress in targeted and immunotherapies, which offer advantages over systemic chemotherapy, there are still limitations in current treatments for metastatic CRC (3): Patients with microsatellite stable tumors (5), lack of biomarkers to predict the response to immunotherapy and so on. This review focuses on the roles of immune cells and molecules in CRC metastasis, particularly exploring the mechanisms of immune tolerance and evasion. We aim to evaluate the potential and limitations of these components in clinical applications, highlighting the need for novel therapeutic strategies to overcome immune evasion in metastatic CRC, and to foste r future research in this vital area.

## 2 The role of immune cells in mediating the metastasis cascade of CRC

In the progression of colorectal cancer (CRC), the immune system is fundamentally instrumental (6). The tumor microenvironment (TME), encompassing immune cells, fibroblasts, endothelial cells, and matrix proteins, significantly influences tumor growth and metastatic spread, impacting processes like seeding, proliferation, and evasion of immune response. Key players within the TME include tumor-associated macrophages (TAMs), CD4+ T (helper T) cells, dendritic cells (DCs), regulatory T cells (Tregs), and tumor-associated neutrophils (TANs), each playing a vital role in CRC’s metastatic journey.

### 2.1 Tumor-associated macrophages

**Figure 1:**
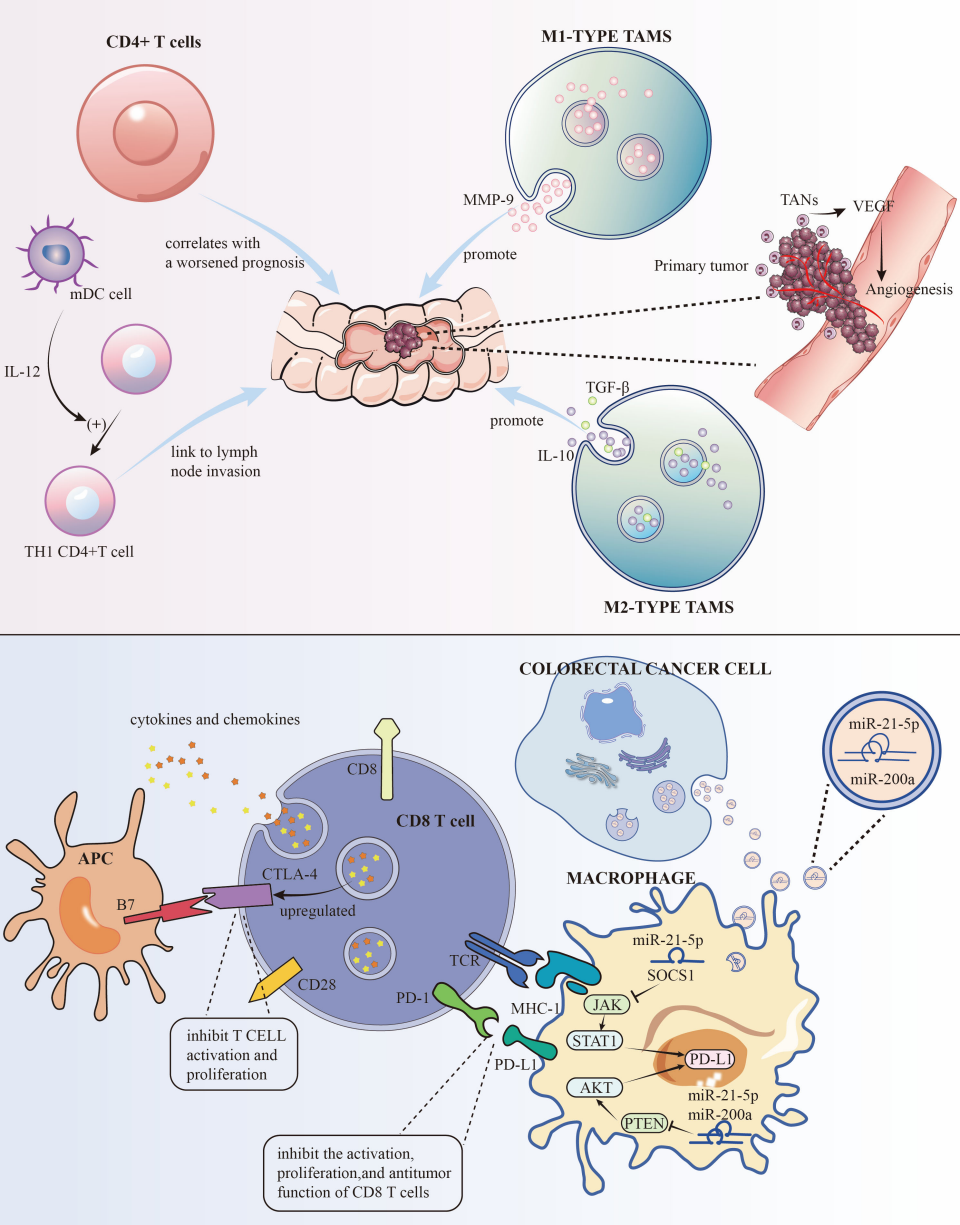
The roles of some immune cells to the development of CRC and the mechanisms of PD-L1 and CTLA-4 for immune evasion.

Traditionally, TAMs are divided into “M1” and “M2” types, which are associated with pro-inflammatory, immune-activating, anti-tumor properties, and immune-suppressive, tumor-promoting properties, respectively (7). M1 type produces pro-inflammatory and immune-activating cytokines like IL-1β, IL-6, and TNF-α. While generally seen as anti-tumor, their effectiveness depends on the environment. For instance, in colitis models, M1 type TAMs can promote chronic inflammation, increasing the risk of CRC. Studies have shown that M1 type TAMs can promote tumor invasion and metastasis by secreting factors like MMP-9. Conversely, M2 type TAMs accelerate tumor progression and invasion by secreting immune-suppressive factors like IL-10 and TGF-β (**Figure 1**, **Table 1**). Understanding the dual roles of TAMs opens avenues for targeted therapies. Modulating the balance between M1 and M2 phenotypes may offer a strategy to harness the anti-tumor potential of TAMs, potentially influencing treatment outcomes.

**Table 1:** The different aspects of immune response-related molecules in CRC metastasis.

Category	Role/Function in CRC Metastasis	Key Points/Findings	References
**Tumor-Associated Macrophages (TAMs)**	Mediate tumor growth and metastasis	M1 type: pro-inflammatory, can promote/inhibit tumor; M2 type: immune-suppressive, promote tumor progression	(7)
**CD4+ T Cells**	Influence hepatic metastasis	TH17 subset linked to worsened prognosis after surgery	(8)
**Dendritic Cells (DCs)**	Antigen presentation, immune response initiation	Myeloid DCs predominant in CRC; link to lymph node invasion	(9, 10)
**Regulatory T Cells (Tregs)**	Immune suppression within TME	Associated with heightened metastasis and poorer outcomes	(11–14)
**Tumor-Associated Neutrophils (TANs)**	Promote tumor invasion and metastasis	Involved in angiogenesis regulation, resistance to VEGF inhibitors	(15, 16)
**Cytokines (e.g., IL-17, TNF-α, IL-6)**	Modulate tumor growth and immune response	Both pro-inflammatory and anti-inflammatory cytokines play roles in CRC pathogenesis	(28–32)
**Cytokines (IL-17A, IL-17F, and IL-22)**	Display anti-tumor effects in CRC	Aiding immune cell recruitment, tissue repair, and reducing inflammation and angiogenesis-promoting factors (IL-17A has a dual role in tumor development)	(36)
**Immune Evasion Mechanisms (PD-1, PD-L1, CTLA-4)**	Inhibit immune cell activation and response	High PD-L1 levels in metastases; TGF-β impairs DCs; CTLA-4 inhibits T cell activation	(44, 46–49)
**Emerging Immunotherapies**	Target immune checkpoints, enhance anti-tumor immunity	PD-1 inhibitors (e.g., nivolumab) show promise, especially in MSI-H/dMMR CRC cases	(57–59)

### 2.2 Helper T

Cells CD4+ T cells, particularly the TH17 subset, have been linked to the hepatic metastasis of colorectal cancer (CRC), also known as CRLM. Research has shown that an increase in these cells after surgery correlates with a worsened prognosis (8) (**Figure 1**, **Table 1**). Strategies aimed at modulating these cells may hold promise in improving post-surgery outcomes.

### 2.3 Dendritic cells

As crucial antigen-presenting cells, DCs play a pivotal role in the immune response. They capture and present external antigens to T cells. Within CRC, it’s often myeloid DCs that predominate. These cells are integral in cell-mediated immunity and in nudging naïve CD4+ T cells towards a TH1 phenotype. They are also noted for their presence at the tumor’s invasive edge and their link to lymph node invasion (9, 10) (**Figure 1**, **Table 1**). Strategies that maintain the balance of DCs(Lymophoid DCs and myeloid DCs) and T cells(TH1 and TH2) could be explored as a potential adjunct to existing treatments, aiming to improve outcomes, particularly in lymph node-involved cases.

### 2.4 Regulatory T cells

In the tumor microenvironment (TME), Tregs are key players in immune suppression. Their presence is often tied to heightened metastasis and poorer outcomes in various cancers (11). In CRC, the role of Tregs in the TME is multifaceted, with indications of their dual functionality (12–14) (**Table 1**). Understanding the nuanced role of Tregs in CRC provides insights for targeted interventions. Modulating Treg activity within the TME may be a viable strategy to disrupt immune evasion mechanisms and improve treatment responses.

### 2.5 Tumor-associated neutrophils

The link between TANs and patient prognosis is a growing research focus. These cells are implicated in promoting tumor invasion, partly through mechanisms like angiogenesis regulation (**Figure 1**) and increasing tumor cell resistance to VEGF inhibitors. They also contribute to metastasis by accelerating the breakdown of the basement membrane (15, 16) (**Table 1**). Strategies aimed at regulating TAN functions may enhance the efficacy of existing therapies, especially those targeting angiogenesis.

### 2.6 Other cells in the tumor microenvironment

Beyond immune cells, the TME comprises stromal cells, the extracellular matrix (ECM), cancer-associated fibroblasts (CAFs), and endothelial cells (ECs), all playing roles in metastasis (17). The ECM, made up of structural proteins such as collagen and proteoglycans, undergoes remodeling by TME cells, affecting both its structure and function (18). It serves not just as a scaffold for tumor cells but also in roles like intercellular adhesion and paracrine signaling, contributing to tumor growth, immune evasion, and metastasis (19–23). CAFs are crucial for ECM upkeep, fibrosis, angiogenesis, immune suppression, invasion, and chemoresistance. The vascular system, composed of ECs, peripheral cells, smooth muscle cells, and progenitor cells, plays a significant role in CRC invasion and therapy targeting (24). The liver is often the primary metastasis site in CRC (25), with interactions between tumor and liver cells, liver sinusoidal endothelial cells (LSECs), and others fostering CAF formation and enhancing cellular stemness and epithelial-mesenchymal transition via exosomes (26, 27). Comprehensive approaches targeting various components of the TME, including ECM, CAFs, and vascular elements, may be crucial. Strategies aimed at disrupting these interactions could potentially hinder metastasis and improve the efficacy of CRC treatments.

## 3 Immune-related molecules in colorectal cancer

Colorectal cancer (CRC) is significantly influenced by the TME, where tumor cells interact intricately with various stromal cells. This interaction is facilitated by a range of soluble elements like cytokines, growth factors, chemokines, and components of the extracellular matrix. In CRC’s pathogenesis, cytokines are particularly crucial. Chronic inflammation, for example, often leads to CRC, exacerbated by the dysregulated expression of cytokines.

CRC exhibits a rise in both pro-inflammatory and anti-inflammatory cytokines, which are deeply intertwined with the progression and potential outcomes of the disease. The pro-inflammatory cytokine, IL-17, for instance, not only intensifies tumor-caused inflammation but also assists cancer cells in evading immune detection (28) (**Table 1**). The IL-23/IL-17 pathway’s role in fostering colorectal tumor development is also notable (29). Further, high levels of IL-23, its receptor, and IL-17A correlate with unfavorable prognosis and swift metastatic progression in CRC cases (30).

Cytokines like TNF-α, IL-1β, and IL-6 are pivotal in the intestinal response to inflammation, fostering intricate cell interactions within the intestinal milieu. These interactions involve cells such as intestinal epithelial cells, paneth cells, macrophages, and goblet cells, all contributing to the chronic inflammation seen in inflammatory bowel diseases like Crohn’s disease and ulcerative colitis. Both TNF-α and IL-6 are implicated in CRC progression, activating oncogenic transcription factors like NF-κB and STAT3 (31, 32).

Moreover, cytokines IL-11, IL-17A, and IL-22 show heightened protein-level expression in CRC, playing a role in the disease’s development in both humans and mice (33–35). IL-17A, IL-17F, and IL-22 display anti-tumor effects in CRC, aiding immune cell recruitment, tissue repair, and reducing inflammation and angiogenesis-promoting factors (36). Interestingly, IL-17A has a dual role in tumor development, whereas IL-17F exhibits tumor-suppressing properties (36) (**Table 1**).

In addition, the CXCL12/CXCR4 axis activation is linked with the spread of colorectal cancer and peritoneal macrophage involvement (37, 38). The TGF-β signaling pathway is also believed to play a crucial role in CRC by governing epithelial-mesenchymal transition (EMT) (39). The intricate interplay of abnormally expressed molecules like cytokines, chemokines, growth factors, and enzymes remodeling the matrix contributes to CRC pathogenesis, ultimately affecting disease outcomes (40–43).

## 4 Immune evasion mechanisms of CRC cells

### 4.1 Cancer cells suppress immune cells and immune molecules

To evade immune detection, cancer cells deploy various strategies to inhibit immune cells and molecules. On the surfaces of activated T cells, B cells, and natural killer cells, PD-1, a transmembrane protein, functions as an immune checkpoint receptor. Its main role is to modulate immune responses, diminishing inflammatory activity, thereby serving as a crucial immune checkpoint. Its ligand, PD-L1, another transmembrane protein, is found on tumor cells, macrophages, and other immune cells. These proteins are part of the immune checkpoint protein family. The interaction between PD-L1 and PD-1 suppresses the activation, growth, and tumor-fighting capabilities of CD8+ T cells, aiding in the tumor’s immune evasion (44). Research indicates that PD-L1 levels are higher in lung (45) and liver metastases (46) of colorectal cancer (CRC) than in primary tumors. Cancer cells also produce TGF-β, which impairs dendritic cells (DCs) (47). CTLA-4, a receptor on T cells, inhibits T cell activation and growth by interacting with B7 molecules (CD80 and CD86) on antigen-presenting cells. In CRC, increased regulatory T cells (Tregs) in the tumor microenvironment may upregulate CTLA-4, which dampens immune responses through high CTLA-4 expression. This upregulation, along with changes in cytokines and chemokines, can promote CTLA-4 expression. Enhanced CTLA-4 expression further impedes anti-tumor immune responses by competing with CD28 for B7 molecules, thereby reducing T cell activation and proliferation (48) (**Figure 1**). This creates an immunosuppressive milieu, aiding tumor immune evasion and survival. Additionally, cancer cells limit cytotoxic T cells (CTL) activity through various mechanisms. For instance, TNF-α in CT26 CRC cells heightens PD-L1 expression in stromal cells, which curbs CD8+ T cell Granzyme secretion (49) (**Table 1**). PD-L1 on tumor and stromal cells binds to PD-1 on T cells, further repressing T cell activation. In colorectal cancer, PD-L1 levels are lower in CRC cells but higher in TAMs, which also hinder the immune response through PD-1 expression and support CRC cell immune evasion by releasing CCL5. Thus, TAMs are key targets in cancer immunotherapy. Advanced stages of CRC, characterized by high PD-L1 expression, are linked with more aggressive disease and poorer survival rates (50). Targeting the PD-1/PD-L1 pathway has become a focal point in immunotherapy. Other cells in the TME also suppress CTL function by releasing TGF-β, which lowers the expression of critical lytic enzymes like perforin, Granzyme, and Fas ligand (51). Furthermore, the CCL5/CCR5 signaling pathway recruits regulatory T cells, effectively suppressing CTLs in CRC mouse models (52). Studies by Wei et al.,2017 demonstrate that high PD-L1 expression in colorectal liver metastasis tumors (CRLMs) and primary tumors correlates with an abundance of CD4+ T cells and CTLs (53). Similarly, Katz et al.,2009 observed that high density of CTLs was positively correlated with a 10-year survival rate after CRLM resection, and in multivariate analysis, a high CD8+/low CD4+ phenotype was significantly associated with better long-term survival (54).

### 4.2 Immune evasion mechanisms of CRC cells involving exosomes

Research indicated (55)that small extracellular vesicles (sEVs) are secreted by CRC cells. These sEVs, when absorbed by macrophages, enhance M2 polarization and PD-L1 expression. This process escalates the population of PD-L1+CD206+ macrophages and diminishes T cell activity within the TME CRC. Subsequent studies have identified miR-21-5p and miR-200a within sEVs as crucial in macrophage regulation. By influencing the PTEN/AKT and SCOS1/STAT1 pathways, these miRNAs foster M2 polarization and PD-L1 expression in macrophages. This leads to a decrease in CD8+ T cell activity and promotes tumor progression (**Figure 1**). Hence, focusing on sEV-miRNAs specific to CRC and PD-L1 in TAMs could emerge as an innovative approach to treat CRC and boost the effectiveness of anti-PD-L1 treatments.

## 5 Emerging field of immunotherapy

In the TME, various immune cells such as tumor-infiltrating lymphocytes, TAMs, and TANs play a role. Immunotherapy offers new opportunities for the treatment of solid tumors, especially therapies targeting immune checkpoints. Although there has been progress in immunotherapy for colorectal cancer, there are still obstacles. In clinical trials, immunotherapeutic drugs for metastatic colorectal cancer generally show moderate effects, especially in patients with microsatellite stable tumors (5). Challenges include the lack of biomarkers to predict the response to immunotherapy, the relationship between tumor mutation burden and treatment response, the association of immune cells in the TME with treatment outcomes, and the expression of suppressive immune nucleic acid toxic molecules (56). Significant results have been achieved in the treatment of colorectal cancer patients with microsatellite instability (MSI-H)/DNA mismatch repair deficiencies (dMMR) using PD-1 inhibitors such as nivolumab and other immune checkpoint inhibitors (57). Tumor-infiltrating lymphocytes, particularly CD8+ T cells, play an important role in anti-tumor activity. However, tumor cells evade immune attack by expressing inhibitory immune checkpoint receptors such as PD-1, CTLA-4, and LAG-3, as well as regulatory T cell immune suppressive molecules like TGF-β and IL-10. In colorectal cancer, the expression of PD-1 and CTLA-4 on T cells and regulatory T cells is associated with a poor prognosis. Therefore, immune checkpoint molecules are potential targets for immunotherapy in colorectal cancer. Blocking immune checkpoints opens a new era in cancer treatment. Targeting immune checkpoints in the tumor microenvironment of colorectal cancer is a novel cancer treatment method that changes the function of immune cells. The response of patients is related to pro-tumoral and anti-tumoral immune cells in the TME (such as TILs, TAMs, and TANs) (58, 59) (**Table 1**). Anti-PD-1, anti-PD-L1, and anti-CTLA-4 are promising immune checkpoint blockers in colorectal cancer. Furthermore, the emergence of new immune checkpoints like TIM-3 and LAG-3, which can inhibit T or NK cell activity (60), is noteworthy. Combining immune checkpoint blockers with other treatments has shown positive results and may become part of successful colorectal cancer treatment. Immune checkpoint blockers have made some progress in cancer treatment, but still face challenges such as treatment resistance and immune toxicity. Therefore, combining them with other treatment strategies like immune adjuvants and tumor vaccines may enhance their effects. In addition, there are numerous potential targets for immunotherapy in colorectal cancer (CRC), such as the IL-33/ST2 axis (61–63), MDSCs (64), and KRAS (65–67). Immune checkpoint blockers in tumor immunotherapy have broad application prospects and provide a new direction for treatment.

Currently, various immunotherapy strategies are under research, including non-specific immunotherapy, specific immunotherapy, and cell therapy. One or more of these strategies are expected to be used in the clinical treatment of colorectal cancer in the future. Developing effective immunotherapy strategies is crucial for addressing colorectal cancer. Future research will focus on immune escape mechanisms, the immune function of lymphocytes, and the impact of individual genetic polymorphisms on treatment response.

## 6 Discussion

The development and metastasis of colorectal cancer (CRC) are closely related to the interactions between immune cells and molecules in the tumor microenvironment (TME). The different immune cells mentioned in this review, such as Tumor-Associated Macrophages (TAMs), CD4+ T cells, Dendritic Cells (DCs), Regulatory T cells (Tregs), and Tumor-Associated Neutrophils (TANs), each play specific roles and mechanisms in the metastasis of CRC. For instance, the dual nature of M1 and M2 macrophages in TAMs in promoting and inhibiting tumor progression highlights the complexity of the immune system in cancer development. Moreover, the role of CD4+ T cells in liver metastasis of CRC and the critical role of DCs in antigen presentation further emphasize the diversity and importance of immune cells in the TME.

The immune evasion mechanisms of colorectal cancer cells are a key area in cancer research. CRC cells evade the immune system’s surveillance and attack through various mechanisms, such as the secretion of sEVs, upregulation of PD-L1 expression, and the action of CTLA-4. These findings not only reveal the complexity of tumor immune evasion but also provide potential targets for developing new immunotherapy strategies.

The development of immunotherapy has brought new hope for the treatment of colorectal cancer. The application of immune checkpoint inhibitors, especially in patients with high microsatellite instability CRC, has shown significant therapeutic effects. However, these treatments have limited effects in patients with microsatellite stable tumors, highlighting the need for more personalized and targeted treatment approaches. Combining immune checkpoint inhibitors with other treatment methods, such as targeted therapy and chemotherapy, may improve treatment effectiveness and reduce drug resistance.

Future research should focus on a deeper understanding of immune escape mechanisms, improving the function of immune cells, and exploring the impact of individual genetic polymorphisms on the response to immunotherapy. Additionally, new therapeutic strategies, such as specific immunotherapies and cell therapies, are expected to play a significant role in the treatment of colorectal cancer. Integrating these aspects of research can not only deepen our understanding of the CRC immune microenvironment but also pave the way for developing more effective treatment strategies.

In summary, the treatment of colorectal cancer is a multifaceted and multilevel challenge that requires the integration of knowledge and technology from different fields. By gaining a deeper understanding of the complex relationship between the immune system and CRC, we can move towards developing more effective and precise treatment methods.

## Author contributions

RH: Conceptualization, Data curation, Writing – original draft. SH: Writing – original draft. JL: Conceptualization, Data curation, Writing – original draft. LS: Writing – original draft. XG: Conceptualization, Writing – original draft, Writing – review & editing. HC: Conceptualization, Supervision, Writing – original draft, Writing – review & editing.
